# Neuroinflammation in HIV-associated depression: evidence and future perspectives

**DOI:** 10.1038/s41380-022-01619-2

**Published:** 2022-05-26

**Authors:** Arish Mudra Rakshasa-Loots, Heather C. Whalley, Jaime H. Vera, Simon R. Cox

**Affiliations:** 1 Edinburgh Neuroscience, School of Biomedical Sciences, The University of Edinburgh, Edinburgh, UK; 2 Lothian Birth Cohorts Group, Department of Psychology, The University of Edinburgh, Edinburgh, UK; 3 Division of Psychiatry, Centre for Clinical Brain Sciences, Royal Edinburgh Hospital, The University of Edinburgh, Edinburgh, UK; 4 Department of Global Health and Infection, Brighton and Sussex Medical School, University of Sussex, Brighton, UK

**Keywords:** Neuroscience, Molecular biology, Depression

## Abstract

People living with HIV face a high risk of mental illness, especially depression. We do not yet know the precise neurobiological mechanisms underlying HIV-associated depression. Depression severity in the general population has been linked to acute and chronic markers of systemic inflammation. Given the associations between depression and peripheral inflammation, and since HIV infection in the brain elicits a neuroinflammatory response, it is possible that neuroinflammation contributes to the high prevalence of depression amongst people living with HIV. The purpose of this review was to synthesise existing evidence for associations between inflammation, depression, and HIV. While there is strong evidence for independent associations between these three conditions, few preclinical or clinical studies have attempted to characterise their interrelationship, representing a major gap in the literature. This review identifies key areas of debate in the field and offers perspectives for future investigations of the pathophysiology of HIV-associated depression. Reproducing findings across diverse populations will be crucial in obtaining robust and generalisable results to elucidate the precise role of neuroinflammation in this pathophysiology.

## Introduction

We are living in a global moment of transition, as AIDS-related mortality declines globally and HIV becomes a manageable chronic infection. Due to advancements in combination antiretroviral therapy (cART), the life expectancy of people living with HIV has begun to approach that of the general population [1, 2], although considerable variability remains among key population subgroups [3, 4]. Of the 1.5 million people that were diagnosed with HIV in 2020, many can expect to lead a full life [5].

There are notable clinical concerns in the management of chronic HIV infection, chief among which is the high risk for a range of mental health issues and cognitive deficits [6, 7]. Long-term HIV infection leads to neurocognitive impairment beyond that which is associated with healthy ageing [8], and distinct from that seen in neurodegenerative disorders [9], which persists in mild forms despite cART [10]. Young people living with HIV, including perinatally infected children, are also vulnerable to these cognitive deficits [11].

People living with HIV also face an alarmingly high prevalence of depression compared to the general population, with 30–50% of HIV+ participants reporting depressive symptoms in some studies [12, 13]. The risk of depression in people living with HIV is estimated to be twice that of the general population [14]. Suicide ideation is also substantially higher in people living with HIV, consistent with the observed rates of depression [15, 16]. Therefore, depressive symptomatology is a widespread and serious concern among people living with HIV.

We do not yet know the precise neurobiological mechanisms underlying HIV-associated depression. Some theories suggest that deficits in serotonergic [17] or dopaminergic [18] pathways might explain this risk of depression. Others argue that HIV-associated depression may be a consequence of inflammatory responses [19]. Inflammation, or activation of the body’s immune responses, has been correlated with depression in the general population [20, 21]. This theory is supported by overlapping somatic features such as sleep disturbance and lethargy (collectively termed “sickness behaviour”) that are shared between inflammation and depression [22]. There is growing support for an inflammatory subtype of depression, since some people with depression experience alleviation of symptoms when administered anti-inflammatory medication [23]. Additionally, there is some evidence to suggest that neuroinflammation—a collection of immune responses in the central nervous system, characterised by microglial activation and release of inflammatory cytokines—might be involved in the aetiology of depression [24]. Given the associations between depression and inflammation, and since HIV infection in the brain elicits a neuroinflammatory response [25], it may be argued that neuroinflammation contributes to HIV-associated depression. However, literature in this field is extremely limited and few studies have attempted to elucidate the relationship between neuroinflammation, depression, and HIV.

The objective of this review is to synthesise and critically appraise existing evidence which suggests that neuroinflammation may be a significant contributor to the high risk of depression amongst people living with HIV. We begin with a brief discussion of the strengths and limitations of various techniques currently in use to measure neuroinflammation in humans. We then describe evidence in the literature that points to independent relationships between HIV, depression, and inflammation, and discuss recent preclinical and clinical studies that offer preliminary evidence for an association between neuroinflammation and depression in HIV. Finally, we propose future directions for research into the contribution of neuroinflammation in HIV-associated depression.

## Measuring neuroinflammation in humans

In order to identify the potential contribution of neuroinflammation in HIV-associated depression, we must first be able to accurately assess degree of neuroinflammation in humans. The most direct way to achieve this would be to assess features such as level of activation of resident immune cells or concentration of neuroinflammatory cytokines in brain tissue using brain biopsies or histopathology [26]. However, brain biopsies are highly invasive and generally cannot be justified in research studies, whereas histopathological analyses in humans are limited by availability of post-mortem brain tissue samples [27, 28]. As a result, to assess neuroinflammation in vivo, indirect markers are generally used. Methodologies for in vivo measurement of neuroinflammation have been extensively reviewed elsewhere [29], and we summarise the most widespread techniques here to aid in the consequent discussion of neuroinflammation in HIV-associated depression.

### CSF and blood biomarkers

In the absence of brain tissue to directly assess glial cell activation or cytokine elevations, cerebrospinal fluid (CSF) and blood are useful tissue samples for indirect measurement of neuroinflammation. Metabolic markers in CSF have been used as an indication of neuroinflammation, since CSF has a direct interface with the brain [30]. Inflammatory cytokines such as interleukin-15 (IL-15) and monocyte chemoattractant protein-1 (MCP-1) in CSF may in fact be useful to characterise neuroinflammatory disease progression [31, 32]. However, there is considerable variability in reported CSF biomarker concentrations even within specific neurodegenerative conditions [33], and CSF biomarkers may not accurately reflect the state of cells in the brain. In addition, lumbar puncture to obtain CSF samples is quite an invasive procedure and may deter participation in studies.

Blood assays are less invasive than CSF assays, but also represent a less direct measure of inflammatory processes in the brain as there is limited exchange of molecules across the blood–brain barrier (BBB). C-Reactive Protein (CRP) is a widely-studied inflammatory cytokine whose serum concentrations have been correlated with depression severity [21]. The cytoskeletal protein neurofilament light (NFL), which is released into CSF and blood as a consequence of neuroaxonal injury, has diagnostic value in many neurological diseases [34]. In addition to being a marker of neuroaxonal injury and neurodegeneration, NFL may be a promising proxy measure of neuroinflammation as well [35–37]. Recent studies suggest that NFL levels in both CSF and plasma correlate with levels of commonly-used biomarkers of neuroinflammation such as CXCL10 and neopterin [38]. Plasma NFL is also correlated with the kynurenine-to-tryptophan ratio and kynurenine metabolites, which are both traditional measures of neuroinflammation. Finally, there are correlations between CSF and serum concentrations of both CRP and NFL [39–41], indicating that blood assays may be a preferable method of measuring inflammation to avoid highly invasive and painful procedures for participants. Taken together, these findings indicate that NFL in blood serum or plasma may be used as an indirect biomarker of neuroinflammation.

In addition to serum levels of inflammatory markers such as CRP (which represent acute inflammation), recent advancements have also allowed us to probe DNA methylation signatures of many markers of peripheral inflammation [42, 43]. These epigenetic signatures represent more stable, longitudinal measures of chronic inflammatory markers and have been calculated using data from epigenome-wide association studies (EWAS) for cytokines such as CRP and BDNF [21, 44–46]. However, DNA methylation signatures for NFL, which has only recently been recognised as a proxy measure of neuroinflammation, have not yet been studied. Calculating methylomic signatures for NFL and studying these in large cohort studies could thus be a meaningful opportunity to indirectly explore aspects of chronic neuroinflammation in humans.

### Neuroimaging biomarkers

Neuroimaging techniques offer a more indirect methodology for assessment of neuroinflammation in human participants than blood or CSF assays [47]. The two most widely-used neuroimaging techniques to measure neuroinflammation are positron emission tomography (PET) and magnetic resonance spectroscopy (MRS). PET involves administration of a radioligand designed to bind to a target protein and emit radioactivity, which may be used to quantify a physiological function [48, 49]. In the context of neuroinflammation, this physiological function of interest is microglial activation, which is assessed using PET radioligands that bind specifically to the mitochondrial 18 kDa translocator protein (TSPO) [50, 51]. TSPO binding is considered a measure of microglial activation and is altered in various psychopathologies [52, 53] but not in healthy ageing [54], providing a sensitive biomarker that can be used to distinguish healthy ageing from neuropsychiatric and neurodegenerative disorders. Numerous TSPO-targeted PET radioligands have been developed in recent years, and the choice of radioligand when assessing neuroinflammation may introduce an additional confounding variable and limit comparisons across studies [55, 56]. Moreover, as PET involves injection of a radioactive tracer, use of this methodology to study neuroinflammation can be ethically contentious, especially for children or participants who are seriously ill [57, 58].

[^99*m*^]Tc-tilmanocept is another neuroimaging radiotracer which may possibly be used to measure neuroinflammation via imaging. This radiotracer binds to the CD206 receptor, which is constitutively expressed on the surface membrane of microglia and macrophages, and thus may be used as a marker of microglial activation [59]. Early studies suggest that in vivo nuclear imaging of tilmanocept can serve as a measure of BBB dysfunction [60]. Since the use of tilmanocept as a potential imaging biomarker of inflammation is fairly new, the studies that have applied this radiotracer in samples of people living with HIV so far have focused on cardiovascular disease [61, 62]. However, as tilmanocept has the potential to serve as a measure of microglial activation and BBB disruption, it may in future be used to assess neuroinflammation and neuropathology amongst people living with HIV.

MRS allows us to non-invasively identify and quantify metabolites in the brain as an additional indirect measure of neuroinflammation [63]. MRS investigates the behaviour of atoms with non-zero spin under an external magnetic field to determine the chemical composition of a localised brain region [64, 65]. Proton MRS (or ^1^H-MRS, targeting hydrogen atoms in brain tissue) is most commonly used in clinical studies to assess neurometabolite concentrations, which are often altered in disease states, including in HIV [66–69]. The neurometabolites myo-inositol (mI) and glutamate/glutamine (Glx) are upregulated in activated glial cells, whereas levels of N-acetyl-aspartate (NAA) and choline-containing metabolites such as glycerophosphocholine and phosphocholine (GPC + PCh) are altered when neuronal membrane integrity is compromised [70–73]. Thus, alterations in mI, Glx, NAA, or GPC + PCh concentrations may be indicative of neuroinflammation. Cerebral metabolite concentrations as measured via MRS also correlate with CSF concentrations of some chemokines amongst people living with HIV, which further supports the use of MRS as a non-invasive tool to measure neuroinflammation in this population [74]. However, due to the ubiquity of hydrogen atoms in the brain, MRS struggles with a very low signal-to-noise ratio and requires substantial signal enhancement, and often requires pre-specified Regions of Interest (ROIs) [75].

The advantages and disadvantages of the most commonly-used methodologies to measure neuroinflammation in humans discussed here are summarised in Table 1.

**Table 1:** Strengths and limitations of commonly-used techniques to measure neuroinflammation in humans in the context of HIV.

Technique	Example studies	Strengths	Limitations
Neuropathology	Smith et al. [26] Anthony et al. [223]	Cellular and molecular features of neuroinflammation such as microglial ramification or activation may be observed and quantified directly	Limited availability of post-mortem human brain tissue
Cerebrospinal fluid markers	Cheng et al. [224] Guha et al. [225]	Concentrations of inflammatory cytokines circulating in the central nervous system may be determined directly	High variability; lumbar puncture is an invasive and painful procedure
Blood biomarkers	Lyons et al. [226] Wada et al. [227]	Venepuncture for sample collection is a routine procedure; levels of some blood biomarkers are correlated with CSF levels	Less direct measure of CNS inflammation as there is limited exchange of molecules across the blood–brain barrier
PET Imaging	Garvey et al. [228] Vera et al. [97]	In vivo measurement of microglial and/or astroglial activation in the brain	Heterogeneity in choice of radioligands and analysis; invasive and with potential risks with injection of radioactive tracers
MRS Imaging	Graham et al. [69] Cysique et al. [229]	Non-invasive, relatively straightforward to administer; can detect changes in a range of neurometabolites in localised brain regions	Low signal-to-noise ration; requires pre-specific Regions of Interest (ROIs)

Studies employing these techniques to assess neuroinflammation in HIV are included as examples.

## Pieces of the puzzle

### HIV and (neuro)inflammation

HIV is, at its core, an inflammatory condition [76]. When HIV is first detected in the body, a range of innate immune responses are activated to defend the body, including elevations in circulating cytokines and activation of Natural Killer (NK) cells [77]. These systemic changes represent features of HIV-induced inflammation [78, 79]. Since HIV targets CD4+ T cells, which are crucial for mounting an immune response in the human body, the inflammatory response to HIV is paradoxical in that level of immune activation is positively correlated with disease progression [80, 81]. This inflammation predisposes people living with HIV to a variety of physiological effects, such as cardiovascular disease [82], renal function alterations [83], increased gut permeability [84], and clusters of illnesses collectively termed “inflamm-aging” [85]. In addition to physical health effects, the inflammation elicited by chronic HIV infection has also been linked to cognitive impairment [86].

One of the key reservoirs for latent HIV infection is the brain [87], so it is unsurprising that HIV infection also elicits a neuroinflammatory response [88, 89]. Even though HIV cannot directly infect neurons, its viral proteins (especially gp120 and tat [90, 91]) are known to induce neuronal cell death. Even non-replicating, latent proviral HIV DNA can be transcribed and translated into these neurotoxic viral proteins, which trigger inflammatory responses in the brain [92, 93]. Activation of the NLRP3 inflammasome, triggered by HIV infection and leading to the production of pro-inflammatory cytokines IL-1β and IL-18, is implicated in HIV disease progression and depletion of CD4+ T cells [94, 95]. This inflammasome activation in microglia has been shown to constitute an early and integral aspect of HIV-associated neuropathology, suggesting that HIV induces a neuroinflammatory response that may be detrimental to the central nervous system (CNS) [96].

There are three lines of evidence supporting the theory that HIV infection is linked to neuroinflammation. First, in vitro studies indicate that exposure to HIV viral proteins leads to microglial activation, a hallmark feature of neuroinflammation [25]. These findings are corroborated by clinical investigations which found that people living with HIV show global increases in microglial activation compared to negative controls [97]. Second, HIV infection induces changes in neurometabolite concentrations consistent with inflammation in several brain regions [68, 69, 98]. Finally, concentrations of NFL in both blood and CSF have been shown to be elevated in people with HIV [99–101], indicating neuronal damage, and these levels are reduced when participants start receiving cART [102]. Together, these results strongly suggest that HIV induces an neuroinflammatory response in humans.

HIV is a genetically diverse pathogen, with several subtypes and recombinant variants, including Subtype B (most common in Europe and Americas) and Subtype C (prevalent in southern Africa and India) [103]. Subtype C contains a “defective” Tat protein and has thus been hypothesised to be less neuropathogenic than Subtype B [104]. However, recent studies have shown that the (neuro)inflammatory responses elicited by these two subtypes of HIV do not differ significantly, with a wide range of CSF and serum inflammatory chemokines and cytokines being significantly elevated in individuals with Subtype B or Subtype C HIV compared to HIV-negative controls, but with no difference in the biomarker levels between the two subtypes [105]. On the contrary, an in vitro study testing inflammation and BBB dysfunction in response to Tat proteins from the HIV Subtype B and recombinant HIV CRF02_AG (prevalent in West Africa) found that Tat.B elicited a greater inflammatory response in comparison to Tat.AG [106]. Together, these results suggest that while Subtype C may not be less neuropathogenic than other subtypes as previously hypothesised, there may still be subtype-dependent variability in neuroinflammatory responses which warrant further investigation.

### HIV and depression

Depression has long been known to be the most common neuropsychiatric condition associated with HIV infection [107]. Studies consistently find prevalence rates of about 30% for depression amongst people living with HIV [108], and large-scale meta-analyses estimate that this prevalence rate is twice that in the general population [14]. Amongst people living with HIV, this risk for depression seems to be somewhat dependent on sex [109], such that women are at higher risk for severe or moderate depression whereas men are at higher risk for moderate depression, and on time passed since diagnosis [110], such that individuals who have been diagnosed more recently are at higher risk for depression. Additionally, prevalence rates vary by geography and are substantially higher in the Global South than in the Global North [111], with estimated rates of 40% or higher for countries such as South Africa [112]. Combined with the fact that healthcare and research funding is concentrated in the Global North [113], this severely exacerbates the burden of depression amongst people living with HIV in the Global South.

The potential mechanisms underlying HIV-associated depression have been extensively reviewed elsewhere [19]. Briefly, possible ways in which HIV might render people susceptible to depression may include neurotoxicity leading to neurotransmitter dysfunction, metabolic dysfunction, and chronic elevations in inflammatory cytokines. Some studies have found that changes in immunometabolism, such as the ratio of kynurenine-to-tryptophan (KYN/TRP), are linked to depression amongst people living with HIV [114]. This is particularly important as ART-induced improvements in depressive symptoms seem to be mediated in part by the reversal of tryptophan catabolism [115]. There are significant overlaps between these various hypothesised mechanisms behind HIV-associated depression, for instance, as inflammatory cytokines may induce glial cell activation and an increase in tryptophan metabolism, which may further cause serotonin depletion and elicit overexpression of metabolites such as quinolinic acid [116]. Therefore, the high risk of depression amongst people living with HIV may stem from a combination of dysfunction in immunometabolism, inflammation, and neurotransmitter cascades.

Interestingly, concentrations of CSF HIV RNA, but not plasma HIV RNA, seem to predict onset of depressive symptoms [117]. This suggests that it may be necessary for HIV to interact directly with aspects of the central nervous system—whether this is metabolism, inflammation, or neurotransmitters—in order to elicit depressive behaviours, and that viral load outside the CNS may not be sufficient to explain HIV-associated depression. However, the directionality of this relationship is unclear: depressive symptoms may conversely increase susceptibility to HIV (by increasing risky behaviours) and are known to predict worse clinical outcomes in people living with HIV [118, 119]. Moreover, this relationship is further complicated when considering that antiretroviral medication may affect metabolism and inflammation [120]. Latest-generation ART, while less neurotoxic, is still associated with metabolic alterations that may contribute to chronic inflammation and immune dysfunction, which ultimately may lead to depression [121]. Conversely, while ART normalises the levels of some inflammatory cytokines in people living with HIV, concentrations of other cytokines such as IL-6 and CRP (which are strongly linked to depression) remain elevated [122, 123]. In all, the relationship between HIV and depression is multi-faceted, but seems to be dependent (at least in part) on the direct interaction of HIV with the CNS and independent of ART-induced normalisations in metabolism or inflammation.

It is important to note here that a purely neurobiological explanation will likely not be able to account for the alarmingly high risk of depression in HIV, as the aetiology of depression is complex and multi-faceted. The role of psychosocial and individual factors cannot be neglected, especially as people living with HIV are likely to experience housing or financial insecurity, discrimination on the basis of gender or sexual identity, intimate partner violence, or external and internalised stigma, all of which can contribute to depression [124, 125]. People living with HIV may also receive poor social support and experience social isolation, which may further contribute to increased inflammation and behavioural deficits [126, 127]. Moreover, some people with depression (including people living with both HIV and depression) do experience symptom relief in response to non-pharmacological interventions such as psychotherapy and social support [128], further indicating that psychosocial factors play a critical role in the progression of HIV-associated depression. Nevertheless, given that depressive symptoms do have strong biological correlates and predict negative clinical outcomes with regards to HIV, investigating the neurobiological pathogenesis of HIV-associated depression is essential in improving quality of life for people living with HIV.

### Depression and (neuro)inflammation

Identifying the pathophysiology of depression is crucial to designing effective, targeted therapeutic interventions for treatment. For several decades, the prevailing theory in biological psychiatry was that depression stems from a dysfunction in serotonergic pathways [129]. This theory was supported by serendipitous clinical findings that pharmacological interventions such as monoamine oxidase inhibitors could alleviate the symptoms of depression in some patients [130]. Dysregulation of serotonin and other neurotransmitters has been strongly implicated in depression over the years [131, 132]. However, the neurotransmitter theory of depression struggles with certain key challenges, including that a third of patients do not respond to treatment with antidepressants such as selective serotonin reuptake inhibitors (SSRIs) [133, 134]. Given this, and since there is increasing evidence that depression may in fact be a constellation of symptoms, efforts have been made to classify depression into subtypes of varying aetiology, so that interventions may be precisely targeted to individuals instead of a “one-size-fits-all” approach to antidepressant treatment [135, 136].

Since there is considerable overlap between the somatic features of inflammation and depression, there is rising support for the theory that depression (or at least a subtype of depression) may be associated with inflammation [137]. In particular, behavioural changes such as fatigue, motivational anhedonia, and sleep disturbances (“sickness behaviours”) can be observed in both depression and inflammation [138], and depressive symptoms can be alleviated with anti-inflammatory medication [139]. Significant elevations in concentrations of inflammatory cytokines such as IL-1, IL-6, CRP, and Tumour Necrosis Factor (TNF-α) have consistently been observed in people with depressive symptoms [140–143]. A large meta-analysis also found that inflammation is a significant predictor of depression [144]. CRP, especially, has been the subject of much attention as a peripheral marker of inflammation that seems to be correlated with depression incidence and severity [41, 145]. Associations between CRP levels and depression remain significant even after controlling for some clinical and psychosocial factors [146], and elevations in CRP levels predict decreased functional connectivity in key brain regions [147]. While the precise nature of this association remains unknown, it is theorised that this may be a bidirectional relationship [148]. Evidence from a longitudinal twin-study suggests that different inflammatory cytokines might interact with depression differently: elevations in CRP may perhaps be a consequence of depression, while elevations in IL-6 act as a risk factor for depression [149]. Later studies have linked CRP and IL-6 to specific symptoms of depression and identified a potential causal link between IL-6 and depression [150]. NFL, which has recently gained attention as a useful biomarker of neuronal injury and neuroinflammation, has also been associated with depressive symptom severity [151]. Further genetic and immunological investigation is necessary to determine whether CRP, IL-6, NFL, or other inflammatory cytokines may have a causal relationship with depression.

The gut microbiome also interacts significantly with inflammation and immunometabolism to influence depression incidence and severity [152]. Manipulation of the gut microbiome has shown some promise in the treatment of depression, although the mechanisms underlying this “microbiota-gut-brain axis” remain unclear [153]. Recent evidence has shown that gut microbiome dysbiosis is linked to worse psychological well-being amongst people living with HIV and depression in individuals co-infected with HIV and HCV [154, 155]. While further work is necessary, these studies suggest that the gut microbiome may be an important influence in regulating the relationship between HIV and depression.

Strong evidence has been identified in recent years pointing to a link between depression and neuroinflammation [156, 157]. Elevations in neurometabolites such as mI in the brain are associated with depression incidence and severity [158]. CSF concentrations of markers such as TNF-α and IL-1β are also correlated with depression severity [159, 160], and exogenous induction of inflammation can produce some core features of depression [161]. Conversely, administration of minocycline (an anti-inflammatory drug) attenuates depressive symptoms [162], and animal models indicate that minocycline achieves this by inhibiting microglial activation [163, 164]. Indeed, Richards et al. [165] found that TSPO binding (recall that this is a marker of microglial activation) is highest in patients with depression who are *not* on antidepressant medication, whereas medicated participants show comparable TSPO binding to undepressed controls. These results lend support to the theory that microglial activation (a key feature of neuroinflammation) may be targeted by antidepressant therapies for treatment-resistant depression [24]. Taken together, these findings suggest that inflammation, and neuroinflammation specifically, may play a critical role in the psychopathology of depression.

## Putting the pieces together

**Fig. 1:**
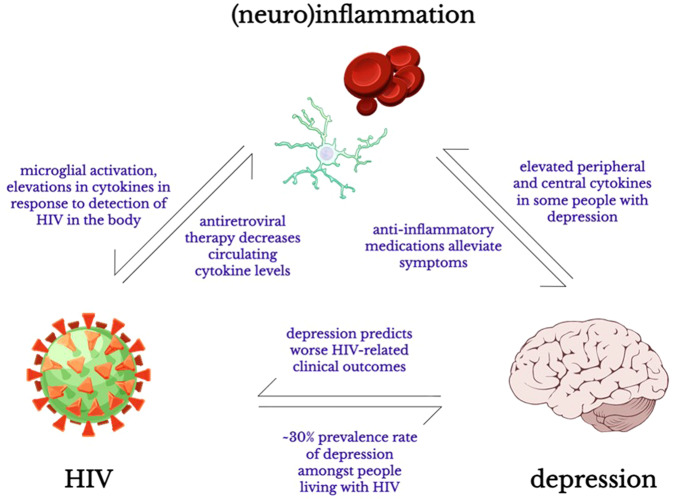
Schematic representation of the relationship between HIV, depression, and (neuro)inflammation. Key pieces of evidence pointing to independent associations between these conditions are summarised. Open source (CC-BY) schematic drawings via SciDraw. Microglia: 10.5281/zenodo.3926033; erythrocytes: 10.5281/zenodo.3926235; human brain: 10.5281/zenodo.3925925; virus: 10.5281/zenodo.3926017.

A substantial body of evidence has shown that independent associations exist between depression, neuroinflammation, and HIV (Fig. 1). However, very few studies have attempted to explore the relationship between all three conditions. The relationship between (neuro)inflammation and depression has been described in great detail in an excellent systematic review by Toenders et al. recently [148]. We point readers particularly to Fig. 3 in the review article by Toenders et al. [148] [p.321] which summarises various proposed mechanisms connecting inflammation and depression, such as metabolic dysfunction including leptin and insulin resistance and hypothalamic-pituitary axis hyperactivity. These physiological alterations have been observed with HIV infection [166, 167], and thus the molecular pathways discussed by Toenders et al. may be highly relevant to HIV-associated depression as well. Therefore, we will not discuss specific molecular or physiological pathways connecting HIV, inflammation, and depression here, and will instead focus on highlighting recent studies that have demonstrated preliminary evidence for such an association amongst people living with HIV specifically.

### Preclinical and post-mortem evidence

Preclinical studies of neuroinflammation in HIV-associated depression present a unique set of strengths and challenges. On the one hand, features of neuroinflammation such as microgliosis may be directly measured in animals using immunostaining of tissue samples [168] or in vivo imaging through cortical windows [169], which is seldom possible in humans as access to primary human brain tissue is not often feasible. On the other, due to the narrow host range of HIV, animal models of HIV infection cannot accurately replicate the progression of HIV infection in humans [170, 171]. Moreover, although animal models of depression have been developed to exhibit many somatic features of depression [172, 173], it remains difficult to study higher-order cognitive and emotional components of depression (such as loneliness or suicidality) in animals. For these reasons, neuroinflammation in HIV-associated depression has not been widely studied in preclinical models.

Advancements in animal models for HIV-associated depression were reviewed by Barreto et al. who suggested that it may be worthwhile to investigate the role of neuroinflammation in these animal models [174]. Since publication of their review, there have been very few attempts to explore this line of enquiry. Nemeth et al. [175] tested whether developmental expression of HIV-related proteins in transgenic rats may be linked to depressive-like behaviours and elevations in neuroinflammatory responses. They found that adolescent HIV Tg rats did exhibit depressive-like behaviours and increased concentrations of MCP-1 (a key neuroinflammatory chemokine) in the hippocampus. Treatment with meloxicam (a selective COX-2 inhibitor) successfully reduced *Mcp-1* gene expression in the hippocampus, but failed to rescue depressive-like behavioural deficits in the HIV Tg rats. Their findings suggest that HIV-related proteins can trigger depressive behaviours in rats, but the expression of *Mcp-1* in the hippocampus may not contribute to HIV-associated depression. The same group later found that the expression of HIV-related proteins during development induced anxiety-like behaviours in Tg rats and hyper-ramification of microglia (theorised to be involved in microglial surveillance and phagocytosis of apoptotic cells [176, 177]), but did not test for depressive-like behaviours in this instance [178]. Taken together, their results suggest that the presence of HIV viral proteins is associated with depressive-like behaviours and neuroinflammatory responses in rats, but whether HIV-induced neuroinflammation elicits these depressive-like behaviours remains unclear. Since research in this area is sparse, it is crucial for future studies to explore the role of neuroinflammation (especially biomarkers other than MCP-1) in animal models of HIV-associated depression and clarify whether blocking specific neuroinflammatory pathways may rescue HIV-related depressive-like behaviours.

Few post-mortem studies have specifically investigated the relationship between neuroinflammatory markers and depressive symptomatology in people living with HIV. One autopsy study did not find any pathological features in post-mortem brain tissues of people with HIV that could predict depression [179], whereas another found that ante-mortem plasma levels of the inflammatory biomarker sCD163 predicted post-mortem observations of microglial activation, but only included one participant with depression in their study sample and thus could not test for associations with depression [180].

### Clinical studies: peripheral inflammatory biomarkers

Clinical investigations of neuroinflammation in HIV-associated depression are limited. The most closely-related studies look at markers such as CRP that may be more closely linked to peripheral inflammation (instead of neuroinflammation), but these are worthy of discussion as they provide some insights into the kind of relationship we may expect between neuroinflammation, HIV, and depression.

An early study of 23 people living with HIV measured plasma concentrations of several inflammatory cytokines (IL-15, IP-10, IL-12, and G-CSF) and found that these cytokines were significantly elevated in HIV+ individuals with depression compared to HIV+ individuals without depression [119]. Plasma cytokine levels and depressive symptom severity were significantly correlated, suggesting that peripheral inflammatory responses may predict depression severity in people living with HIV. Further studies, including a large multi-centre cohort study of 1,727 participants [181], have also observed this correlation between depression and blood biomarkers of systemic inflammation amongst people living with HIV [182, 183]. This evidence supports the hypothesis that people living with HIV may be susceptible to an inflammatory subtype of depression. Since recent evidence suggests that there is significant overlap between systemic inflammation and neuroinflammation in many disease states (contrary to our previous understanding of the CNS as isolated from the peripheral immune system) [184, 185], these trends may perhaps be reproduced when studying neuroinflammation in HIV-associated depression.

While studies generally agree that cytokines such as IL-6 and TNF-α are correlated with depression, the role of CRP (a cytokine that generally exhibits robust correlations with depression [141, 186]) specifically in HIV-associated depression is a major area of debate. Poudel-Tandukar et al. [187] saw a linear relationship between serum CRP levels and depression severity, and Memiah et al. [188] also observed significant differences in CRP levels between HIV+ participants with depressive symptoms and those without. However, several other studies have found no significant association between CRP concentrations and depression amongst people living with HIV [189–192]. (Notably, Ellis et al. [192] did not observe such a correlation for MCP-1 either, which lends support to the preclinical findings discussed previously [175].) In fact, these studies saw significant correlations between depression and other inflammatory markers such as IL-6 [189, 192] and TNF-α [190], but not CRP. Perhaps this indicates that the subtype of depression to which people living with HIV are most susceptible is mediated by other inflammatory cytokines, but not CRP? Inflammation is not the only physiological state in which cytokines such as CRP and IL-6 serve a function, which complicates any inferences we may make about their role in HIV-associated depression [193]. Further studies are necessary to resolve this debate and determine what role, if any, is played by CRP in HIV-associated depression, as this understanding may aid in developing translational tools for subtype-specific screening of depression in people living with HIV.

The inflammatory biomarkers assayed in studies of HIV-associated depression vary considerably, which limits our ability to identify strong candidates with diagnostic or therapeutic value. A recent study [194] investigated associations between levels of six cytokines (IL-1β, eotaxin, IL-15, IL-6, TNF-α, and leptin) and chronic multisite pain (CMP) in a sample of people living with HIV, but also incorporated PHQ-9 scores as a measure of depression in their regression models. They found that only one cytokine—IL-1β—out of the six analytes tested was significantly associated with CMP, and notably, exploratory analyses showed that accounting for total PHQ-9 score reduced the odds ratio of this model. Depression severity and concentrations of the biomarker IL-1β may thus be interlinked amongst people living with HIV. Memiah et al. [188] tested whether “mental health symptoms” correlated with five inflammatory biomarkers: CRP, IL-6, IL-18, soluble tumour necrosis factor receptor-I (sTNFR-I) and soluble TNFR-II. While their primary outcome measure was not specifically depression, the “self-reported mental health symptoms” in this study included many key features of depression. The authors reported that CRP and soluble TNFR-II were significantly associated with self-reported mental health issues amongst people living with HIV. Pala et al. [195] similarly sought to identify biomarkers associated with depressive symptoms amongst people living with HIV, and found that IL-6 concentrations and monocytes correlated significantly with a severe subtype of depression in their participant sample. Notably, Memiah et al. did not find a significant association between IL-6 and symptoms of depression, whereas Pala et al. did observe such a correlation. Another study found that depression, but not HIV status, was associated with the release of IL-6 [196] which aligns with the findings of Memiah et al. and suggests that the relationship between IL-6 and depressive symptoms may be independent of HIV status. Taken together, these initial findings implicate several central and peripheral inflammatory markers in depression amongst people living with HIV, but heterogeneity in results limits synthesis across studies.

### Clinical studies: neuroinflammatory biomarkers

While most studies discussed in the previous section involve a broad panel of central and peripheral inflammatory biomarkers, few published studies have attempted to expressly investigate a relationship between a specific measure of neuroinflammation and HIV-associated depression. Saloner et al. [197] recently compared levels of blood biomarkers of neuroinflammation between people living with HIV and negative controls. In this study, the authors examined the associations of CSF dopaminergic biomarkers with depression severity (measured as Beck Depression Inventory [BDI-II] scores) and neuroinflammation (measured as a composite score of CSF concentrations of sCD14, MCP-1, IP-10, and neopterin) in a sample of HIV+ and HIV− participants [197]. Although their primary research question concerned the relationship between depressive symptoms and CSF dopaminergic markers, they did report a statistically non-significant trend between higher BDI-II scores and higher composite CSF neuroinflammation score [p.4] [197]. They also saw a significant correlation between BDI-II scores and CSF IP-10 concentrations specifically, but this statistical significance did not persist after FDR correction. Crucially, no such correlations were observed in the HIV- group, offering preliminary evidence that the relationship between neuroinflammation and depression may be dependent on HIV infection status.

Woods et al. [198] present the most promising results to date that implicate neuroinflammatory markers in depressive symptoms amongst people living with HIV. In this study of older adults with and without HIV, the authors found that plasma concentrations of brain-derived neurotrophic factor (BDNF) were significantly associated with depression, such that lower levels of plasma BDNF were linked to higher levels of depressive symptoms. These findings are encouraging, but isolated: substantial further work is necessary to determine the association between various neuroinflammatory markers and depression severity in HIV, and to utilise other measures of neuroinflammation (such as neuroimaging) in this direction. It will also be crucial to replicate these findings in samples with distinct demographic profiles, as no effort has yet been directed at determining the interactions, if any, of psychosocial factors with neuroinflammation in HIV-associated depression.

## The way forward

### Clinical studies of neuroinflammation and depression in HIV

Given the strong body of evidence linking neuroinflammation, depression, and HIV, and yet the dearth of studies investigating the relationship between the three, the vital first step towards the future is to conduct clinical studies of neuroinflammation in HIV-associated depression. Through these clinical studies, we may begin to resolve the major areas of debate in the field that have been identified through this critical review:

- Is there a reliable association between peripheral concentrations of CRP and depression amongst people living with HIV?
- Are people living with HIV susceptible to a subtype of depression mediated by specific cytokines (such as IL-6) and not others (CRP), for which we can develop subtype-specific therapeutic interventions?
- Is the relationship between neuroinflammation and depression dependent on HIV infection status, and is there subgroup variability or differences in brain structure and function within this population?

Although research in this field is quite sparse, some efforts are underway. Notably, an ongoing clinical trial recruiting HIV+ participants in Uganda is investigating the potential for group psychotherapy to improve the severity of depression and whether depression is linked to inflammation in this group [199]. Results from this clinical trial will shed further light on the complex interactions between neuroinflammation, depression, and HIV.

A range of research methodologies are available to neurovirologists to measure neuroinflammation in people living with HIV, and these were summarised in this review. We particularly draw the readers’ attention to neurofilament light (NFL) as a blood biomarker of great potential in assessing degree of neuroinflammation. Although NFL is a marker of neuroaxonal injury, given the evidence discussed here which points to strong correlations between NFL and “traditional” biomarkers of neuroinflammation, and since there is considerable overlap between neurodegeneration and neuroinflammation, plasma or serum NFL may in future prove to be a useful indirect measure of neuroinflammation for which sensitive assays are already available. NFL concentrations in blood correlate with CSF levels, indicating a reliable measure of CNS inflammatory responses, and can be measured with an assay that is considerably less painful for participants than lumbar puncture, which may help boost participant willingness to contribute to these studies. Furthermore, calculating methylomic signatures for NFL using EWAS data (as has been done previously for CRP [42, 44]) might allow us to assess chronic elevations in NFL and offer a longitudinal measure of neuroinflammation, provided that we obtain relevant and sufficiently-powered discovery data. These serum and epigenetic NFL markers may allow us to study short- and long-term changes in NFL levels, which, combined with longitudinal data on depression severity and HIV viral loads, may elucidate the relationship between neuroinflammation and depression through the course of chronic HIV infection.

There are considerable differences in methodology, statistical approach, and reporting of effect sizes in the literature in the field. Consider three representative studies of inflammation and cognition amongst people living with HIV. Anderson et al. [102] report a slope of −11.5 for their multivariate mixed-effects model between plasma NFL concentrations and composite cognitive scores, indicating that cognition worsened by 11.5 points for each logarithmic unit increase in plasma NFL. Saloner et al. [183] report an unstandardised regression estimate of −0.025 for their cross-level interaction between plasma CRP and average depression scores on global cognition, which suggests that global cognition worsened by 0.025 points for each unit increase in CRP for participants with a specific average depression score. Imp et al. [200] report a standardised beta weight of −0.30 for their regression model testing associations between plasma sCD163 levels and global cognitive scores, indicating that global cognition worsened by 0.30 points for each unit increase in plasma sCD163 concentration. This heterogeneity in methodology and statistical reporting limits synthesis of results across studies. Variability in reporting effect sizes also restricts our ability to carry out appropriate sample size estimations for future studies. Many studies in the field, especially those involving PET, struggle with small sample sizes due to resource constraints, and accurate power analyses (involving standardised effect sizes) are crucial in ensuring that the conclusions drawn from these studies are placed in the appropriate statistical context. Thus, there is a need to standardise statistical approaches in the field to allow for robust experimental design and meta-analysis of findings.

Therefore, methodological approaches to measuring (neuro)inflammation and reporting of effect sizes in these studies are quite variable. Nevertheless, it is possible to make some estimation of the effect size for this relationship with two principles in mind: (1) our measures of neuroinflammation in humans are indirect and imprecise; and (2) neuroinflammation is only one of several biological and psychosocial factors that may be relevant to HIV and depression. In that light, we would reasonably expect neuroinflammation to have a small contribution to the pathogenesis of HIV-associated depression when correctly estimated. Therefore, going forward, researchers should clearly report standardised estimates of effect sizes and exercise caution when reviewing reports of large effects in this context [201].

Once a link between neuroinflammation and HIV-associated depression has been established through future studies, the exact nature of this relationship may be investigated. It is probable that HIV and depression interact actively with each other and co-morbidity of the two conditions would necessitate more concerted effort than treating each condition [202] If neuroinflammation is indeed linked with depression in HIV, what is the direction and causality of this relationship? It is possible that HIV elicits a neuroinflammatory response, which then leads to depressive symptoms. However, it is equally likely that an HIV infection (through biological or psychosocial pathways) causes depression, which then leads to neuroinflammation. Which pathway represents the true mechanism underlying this association will impact the development of targeted therapeutics: if HIV-associated neuroinflammation causes depression, then we may use anti-inflammatory medication to treat this depression, but if HIV-associated depression manifests as neuroinflammation, then anti-inflammatory medication might not attenuate depressive symptoms. Therefore, it is crucial to determine the precise relationship between neuroinflammation, HIV, and depression.

### Reproducing findings across diverse populations

Some of the incongruity in evidence relating to neuroinflammation, depression and HIV may be attributed to experimental variability, but the role of participant profiles must also be taken into account when interpreting these findings [203]. Social, economic, cultural, and structural factors can confound results and contribute to heterogeneity in findings across studies [204]. Moreover, the burden of depression is not universal, and there is substantial variability in the prevalence of depressive symptoms and accessibility of mental healthcare for certain population subgroups [205, 206]. Finally, replication of findings instills greater confidence in the generalisability of results and aids in effective translation to clinical interventions [207, 208]. For these reasons, future work into the role of neuroinflammation in HIV-associated depression must attempt to replicate findings across diverse populations.

In particular, we recommend that researchers and funding bodies prioritise these investigations in three key populations. First, there is a meaningful opportunity to focus on HIV-associated depression amongst children and adolescents. Not only is there strong evidence for the burden of mental health challenges faced by this population, but exploring neuroinflammation as a specific contributor to HIV-associated depression in longitudinal studies might help us develop distinct neuroinflammatory profiles for healthy development and HIV-induced developmental changes [209, 210]. Indeed, such investigations offer a unique opportunity to determine what effects, if any, perinatal HIV infection has on neuroinflammation, whether these early neuroinflammatory responses can predict risk for depression later in life, and whether there may be a critical neurodevelopmental window for interventions to minimise long-term depression trajectories [40, 69]. Second, participants who use psychoactive drugs should be included in studies of neuroinflammation and HIV. Drugs such as methamphetamine or cocaine may elicit inflammatory responses in the brain [211], and there is overlap between depression risk, drug use, and HIV [212]. However, well-designed preclinical and clinical experiments may distinguish between HIV-associated neuroinflammation and drug-associated neuroinflammation, while helping us understand the interactions between drug use, HIV, neuroinflammation, and risk for depression. Such studies may improve our ability to screen for and treat depression for HIV+ individuals who use drugs, but this is not possible if people who use drugs continue to be automatically excluded from participating.

**Fig. 2:**
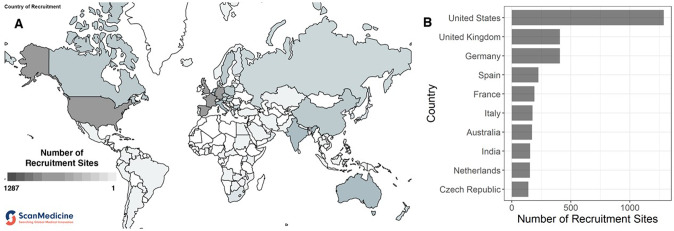
Global distribution of recruitment sites by country for clinical trials investigating HIV and depression. We ran a search for clinical trials catalogued in 11 registries using the keywords “HIV+ depression” in the open source database ScanMedicine. **A** Map of the world with number of clinical trial recruitment sites for HIV-associated depression. Darker shading represents greater number of recruitment sites in that country. **B** Distribution of recruitment sites in the top 10 countries. The 4471 clinical trials that met the search criteria recruited participants across 5619 total recruitment sites, of which 1287 (22.9%) were in the US. Nearly half (*n* = 2518, 44.7%) of all recruitment sites were located in the US, Germany, the UK, Spain, and France. The global distribution of clinical trials of depression and HIV is skewed, with few studies involving participants from the Global South, where there is a substantial burden of HIV-associated depression. World map data visualisation adapted from: ScanMedicine, NIHR Innovation Observatory (NIHRIO), Newcastle University (scanmedicine.com).

Finally, the greatest burden of HIV infection lies in the Global South, where access to latest-generation cART and psychological healthcare is also limited [213, 214]. However, a majority of neurovirology research is carried out in the Global North, with over 80% of research outputs concerning HIV/AIDS being produced by researchers from North America and Europe (González-Alcaide et al. provide an excellent summary of these concerns [215]). These trends are reflected in research specific to HIV-associated depression, as well. We ran a systematic search for all clinical trials in the 11 registries catalogued in the new open source database ScanMedicine using the keywords “HIV+ depression” (https://www.scanmedicine.com/) and examined the distribution of country of recruitment for these trials (Fig. 2). 4471 clinical trials met the search criteria. These trials recruited participants across 5619 total recruitment sites, of which 1287 (22.9%) were located in the US. The top five countries by number of registered clinical trials investigating HIV and depression, representing nearly half (*n* = 2518, 44.7%) of all trials, were the US, Germany, the UK, Spain, and France. In fact, only two countries (India and China) outside of North America, Europe, or Australia were in the top 20 countries by number of trials (see Supplementary Materials). This preliminary data is presented here to illustrate the fact that, given the overwhelming concentration of research funding in affluent nations, these studies often involve participant groups whose physical and mental health, socioeconomic backgrounds, and institutional access may not reflect those of the majority of people who bear the burden of HIV-associated depression (for instance, see ref. [216]).

Moreover, the majority of these studies involve HIV+ participants who are virally-suppressed and receiving cART [181, 217]. Only a few - those primarily recruiting participants in the Global South - include ART-naïve participants [189]. Antiretroviral drugs are known to contribute to neuropsychiatric effects such as sleep disturbances and depressive symptoms [218]. This makes it difficult to ascertain whether inflammatory responses or depressive symptoms observed in these studies arose as a result of HIV infection or ART. Studies involving HIV + individuals who are not yet receiving cART will be essential in delineating the individual contributions of HIV itself and of cART to these neuropsychiatric complications. Since it is difficult to recruit ART-naïve participants in the cART era in the Global North, such studies are more feasible in the Global South. For these reasons, investigations of HIV-associated depression involving participant cohorts in the Global South - especially southern Africa, which is fast becoming a hub for HIV/AIDS research [219]—must remain a focus for funding bodies in order to better understand confounding influences on findings, prioritise global health equity, and improve the generalisability of our findings. Involving participants from these underserved research populations using co-production or participatory research methods may also provide insight into which research questions and outcomes should be a focus for future work and ways to engage stakeholders more broadly [220].

Attempting to reproduce findings across diverse populations will allow us to parse out the complex interactions between neurobiological, psychosocial, and institutional factors that give rise to HIV-associated depression [221]. No single mechanism can sufficiently explain the aetiology of HIV-associated depression, but if we are intentional in our efforts to study these phenomena in populations from distinct clinical, socioeconomic, and cultural backgrounds, we may better understand the individual contributions of each of these factors to this overarching burden of depression. By leveraging this understanding to develop targeted mental health interventions, we may be able to reduce transmission of HIV via “positive prevention” [222]. Therefore, by investigating the risk of depression faced by people living with HIV, we may make substantive progress towards the global goal of HIV/AIDS eradication.

## Conclusion

In this critical review, we synthesised evidence for independent associations between HIV, depression, and (neuro)inflammation. Preliminary preclinical and clinical evidence lends support to the hypothesis that neuroinflammation may play an important role in HIV-associated depression, although data on this relationship is extremely sparse. We also identified some key areas of debate where further work is necessary to reconcile contradictory findings. Future investigations should focus on further characterising this relationship. Studies with robust experimental design that attempt to reproduce findings in key populations must be a priority in order to approach an accurate and generalisable understanding of the contribution of neuroinflammation to HIV-associated depression.

## Supplementary information

ScanMedicine Search Results

## Data Availability

Data from the ScanMedicine clinical trial registry search is available in Supplementary Materials for this paper. No other new data was created or analysed in this project.
